# A Case of Antineutrophil Cytoplasmic Antibodies (ANCA)-Associated Vasculitis Post COVID-19 Vaccination

**DOI:** 10.7759/cureus.23162

**Published:** 2022-03-14

**Authors:** Zaki Al-Yafeai, Benjamin Joesph M Horn, William Terraccaine, Alvin Jose, Prathik Krishnan

**Affiliations:** 1 Internal Medicine, LSU (Louisiana State University) Health Shreveport, Shreveport, USA; 2 Anaesthesia, LSU (Louisiana State University) Health Shreveport, Shreveport, USA; 3 Emergency Medicine, LSU (Louisiana State University) Health Shreveport, Shreveport, USA; 4 Pulmonary and Critical Care Medicine, LSU (Louisiana State University) Health Shreveport, Shreveport, USA

**Keywords:** canca/proteinase 3-positive granulomatosis with polyangiitis, covid-19, wegener's granulomatosis, pr3-positive granulomatosis with polyangiitis, granulomatosis with polyangiitis (gpa), pr3-anca, covid-19 vaccine, anca associated vasculitis

## Abstract

In this report, we examine the case of a patient who developed antineutrophil cytoplasmic antibody (ANCA)-associated vasculitis after receiving the Pfizer-BioNTech coronavirus disease 2019 (COVID-19) vaccine. This is a case of a 62-year-old female who received the first dose of COVID-19 vaccine in July 2021 before presenting a few weeks later with migrating polyarthralgia and hemoptysis. Autoimmune workup was positive for ANCA against proteinase 3 (PR3).

## Introduction

As the coronavirus disease 2019 (COVID-19) pandemic caused by severe acute respiratory syndrome coronavirus 2 (SARS-CoV2) continues and novel variants arise, there is a pressing need to complete COVID-19 vaccination to prevent future outbreaks. While these novel messenger ribonucleic acid (mRNA) and viral vector vaccines have undergone large clinical trials and are shown to be safe and effective, side effects may occur like other routine vaccines [1]. However, other rare and more serious adverse side effects have been recorded during post-marketing surveillance.

antineutrophil cytoplasmic antibody (ANCA)-associated vasculitis (AAV) are a group of small vessel vasculitis that is associated with the formation of autoantibodies against cytoplasmic components of neutrophils, such as proteinase 3 (PR3)-ANCA and myeloperoxidase (MPO)-ANCA. The associated small vessel vasculitis pathologies include granulomatosis with polyangiitis (GPA), eosinophilic granulomatosis with polyangiitis, and microscopic polyangiitis [2,3]. There have been reports of an association of developing new-onset AAV following the Influenza vaccine [4] and COVID-19 vaccines [5]. In this report, we discuss a case of AAV manifesting after receiving the COVID-19 vaccine.

## Case presentation

A 62-year-old Caucasian female with a past medical history consistent with Hashimoto’s disease presented to an outside facility for concern of multiple episodes of hematemesis. Of note, she initially had presented with intermittent and migrating polyarthralgia alone, until over a course of the preceding weeks she began to have new-onset hematemesis. It was noted that she had received her Pfizer-BioNTech COVID-19 vaccination (Pfizer Inc., New York, United States; BioNTech SE, Mainz, Germany) one month prior to developing symptoms. It was at this point that she decided to go to the hospital.

**Figure 1:**
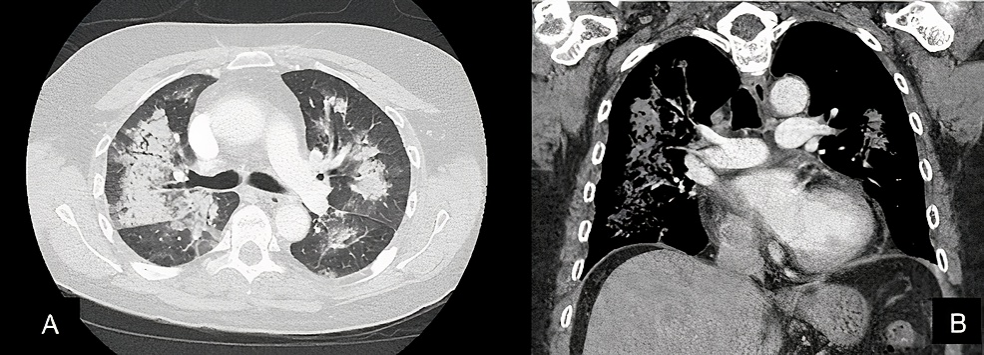
A-B. CT chest displaying diffuse consolidation throughout lung fields.

Upon presentation, she was taken for an esophagogastroduodenoscopy (EGD), displaying pooled blood within her stomach with no clear source of bleeding. A computer tomography (CT) chest displayed bilateral consolidations involving all lobes; peripheral lung fields were spared (Figure 1 A-B). Urinalysis (UA) revealed microscopic hematuria. Further autoimmune workup was begun at this time per recommendations.

On hospital day three, the patient began to have worsening hypoxia and she was transferred to the intensive care unit (ICU). She was placed on a high-flow nasal cannula; however, given continued respiratory distress, she was intubated and placed on a mechanical ventilator. Bronchoscopy showed diffuse alveolar hemorrhages bilaterally. Sequential lavages demonstrated frank blood. She was given three doses of IV immunoglobulin (IVIG), IV methylprednisolone 1g for three days and to be tapered down accordingly (500 mg for three days, tapered to 125 mg every eight hours for three days, and then 60 mg for the final day).

Autoimmune panel was significant for positive PR3-ANCA (>100 IU/mL; reference 4-100 IU/mL) and negative MPO (<9 IU/mL; reference 9-100 IU/mL), anti-glomerular basement membrane (GBM), anti-double-stranded DNA (dsDNA), C3 (136 mg/dL; reference 75-175 mg/dL), C4 (14.6 mg/dL; reference 14-40 mg/dL). Rheumatology was consulted at this time and started on rituximab (375 mg/m2 x 1.81 m2) at 679 mg for four total doses. She was also given one dose of cyclophosphamide (7.5 mg/kg due to worsening glomerular filtration rate (GFR) status) at 620 mg per Nephrology recommendations.

On hospital day seven, the patient began to have worsening renal function given elevated serum creatinine at 3.01 mg/dL up from 1.58 mg/dL the day prior, thus hemodialysis was initiated. Additionally, she was started on broad-spectrum antimicrobials (linezolid, meropenem, micafungin) for a plan of ten total days for possible infectious etiology. However, infectious workups were negative for galactomannan, Fungitell® (Associates of Cape Cod, Inc., Massachusetts, United States), Blastomyces antigen, and blood cultures; thus, de-escalation of antimicrobials was begun. Though initial tests were negative for hemolysis, on day 14, labs were significant for hemolysis including haptoglobin <8 mg/dL, LDH 1809 U/L, and critical hemoglobin of 6.1%, down from 7.2% the day prior. She received multiple sets of transfusions thereafter. Hematology recommended initiating plasma exchange for which she was transferred to a higher acuity-care facility that would be able to provide the intervention.

**Figure 2:**
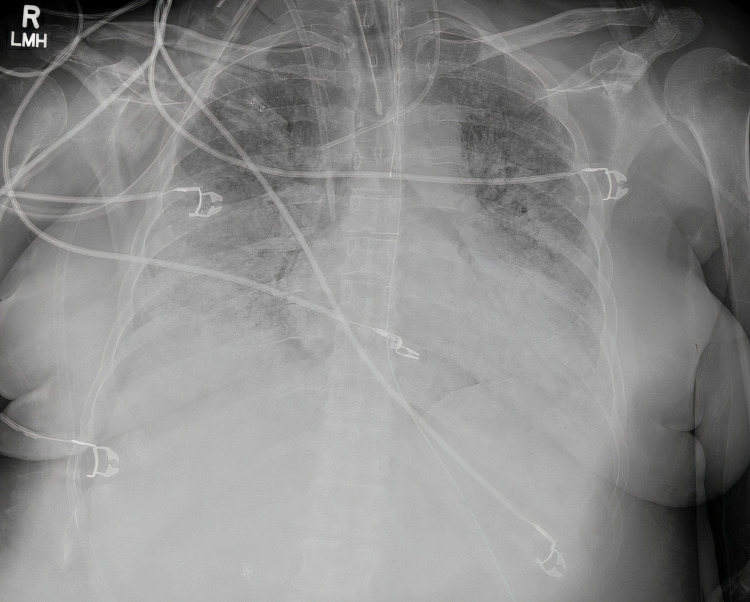
Initial chest x-ray obtained at tertiary care facility displaying bi-basilar opacities prominent within right lung fields.

At the tertiary care facility ICU, she was transferred to the hospital intubated and placed back on a ventilator given the inability to extubate. She presented with the following vital signs: heart rate of 99 per minute, blood pressure of 122/61 mmHg, temperature of 98.1F, respiratory rate of 14 per minute, and SpO2 of 97%. Arterial blood gas (ABG) showed pH of 7.24, pCO2 68.7 mmH, and pO2 83 mmHg. Repeat imaging was obtained. Chest x-ray showed bi-basilar opacities of the right lung (Figure 2).

**Figure 3:**
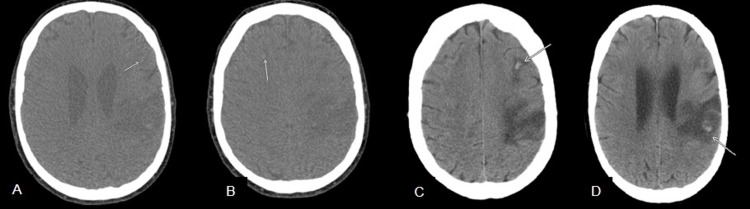
A-D. Initial CT head obtained at tertiary care facility displaying multiple foci hemorrhages within bilateral frontal and right temporal lobe. Arrows indicate multiple foci of hemorrhage.

During her initial days at the tertiary care facility, her neurological status continued to decline. CT of the head without contrast was obtained, for which it was significant for multiple foci hemorrhage within bilateral frontal and right temporal lobes as well as in the right lower lobe with surrounding cerebral edema. CT was also significant for a large hypodensity within the left frontoparietal lobes at the central sulcus with an internal focus of hemorrhage present (Figure 3 A-D). 

In-house Rheumatology and Pathology services were consulted for continued management with plasmapheresis. The patient received a total of seven rounds of plasmapheresis. She was also placed on a rituximab versus cyclophosphamide in ANCA-associated vasculitis (RITUXVAS) trial whereby she received four total doses of rituximab (375 mg/m2 x 1.81 m2) at 679 mg and two doses of cyclophosphamide (7.5 mg/kg due to worsening GFR status) at 620 mg. By the end of her admission in the ICU, repeat biochemical and radiographic evaluation showed that hemolysis and pulmonary hemorrhage improved; however, the patient's neurological function stayed the same. Upon conclusion of the clinical regimen, she was approved for further rehabilitation and management at an outside facility.

## Discussion

Vaccines play a major preventive role in human health. However, due to vaccine-associated immunological responses, generalized side effects emerge such as body ache and headache. Recently, FDA approved three different vaccines for COVID-19. To our knowledge, here we report the first case of PR3-associated vasculitis involving the brain, lungs, and kidneys, after Pfizer-BioNTech COVID-19 mRNA vaccination. Since the COVID-19 vaccination initiative began in December 2020, few cases of COVID-19 vaccine-associated AAV have been reported. Three cases of MPO-ANCA [6-8] and three cases of PR3-ANCA-associated AAV [5,9,10] post COVID-19 vaccination have been described. Interestingly, most of these cases described only renal limited disease manifestations instead of a systemic clinical picture as appears to be described here. 

Although an association between vaccines, infections, and AAV has been previously described as highlighted above, the underlying mechanisms are poorly characterized [11-13]. Studies have suggested that vaccines contain adjuvant molecules that may provoke autoimmune responses. Additionally, vaccine antigens could trigger an autoimmune response via molecular mimicry [14-17]. Arguably, vaccine-associated AAV is more widely studied with the influenza vaccines. These vaccines have been linked to new-onset cases of MPO-ANCA and PR3-ANCA development. In vitro, influenza and rabies vaccines-containing RNA have been shown to trigger PR3-ANCA production [4]. These observations could implicate RNA-based vaccines and AAV. 

It should be noted that this study describes a temporal relationship between the COVID-19 vaccine and AAV. Furthermore, RNA-driven immune responses are poorly characterized. While immune responses elicited by the vaccines might trigger autoimmune processes in certain predisposed individuals, it is important to notice that our patient may have undiagnosed AAV that coincidentally manifested after the COVID-19 vaccination. For example, a prior study showed an association between Hashimoto’s disease and AAV [18], which may have likely placed our patient at risk of AAV after the COVID-19 vaccination. Finally, it is important to emphasize that COVID-19 vaccines are a powerful tool to fight the pandemic and such cases should not affect the decision to be vaccinated.

## Conclusions

In conclusion, our study here highlights a case of AAV after the COVID-19 vaccine. Given that our patient had a prior autoimmune disease history consistent with Hashimoto’s disease, it is likely that the relationship between COVID-19 vaccination and AAV is temporal in nature. While other cases have described a possible association between COVID-19 vaccination and AAV, there is not enough evidence to indicate COVID-19 vaccines in AAV pathogenesis. 
